# Distant Homology Modeling of LCAT and Its Validation through *In Silico* Targeting and *In Vitro* and *In Vivo* Assays

**DOI:** 10.1371/journal.pone.0095044

**Published:** 2014-04-15

**Authors:** Cristina Sensi, Sara Simonelli, Ilaria Zanotti, Gabriella Tedeschi, Giulia Lusardi, Guido Franceschini, Laura Calabresi, Ivano Eberini

**Affiliations:** 1 Laboratorio di Biochimica e Biofisica Computazionale, Università degli Studi di Milano, Milano, Italia; 2 Centro Enrica Grossi Paoletti, Dipartimento di Scienze Farmacologiche e Biomolecolari, Università degli Studi di Milano, Milano, Italia; 3 Dipartimento di Farmacia, Università Degli Studi di Parma, Parma, Italia; 4 Dipartimento di Scienze Veterinarie e Sanità Pubblica, Università degli Studi di Milano, Milano, Italia; Bioinformatics Institute, Singapore

## Abstract

LCAT (lecithin:cholesterol acyltransferase) catalyzes the transacylation of a fatty acid of lecithin to cholesterol, generating a cholesteryl ester and lysolecithin. The knowledge of LCAT atomic structure and the identification of the amino acids relevant in controlling its structure and function are expected to be very helpful to understand the enzyme catalytic mechanism, as involved in HDL cholesterol metabolism. However - after an early report in the late ‘90 s - no recent advance has been made about LCAT three-dimensional structure. In this paper, we propose an LCAT atomistic model, built following the most up-to-date molecular modeling approaches, and exploiting newly solved crystallographic structures. LCAT shows the typical folding of the α/β hydrolase superfamily, and its topology is characterized by a combination of α-helices covering a central 7-strand β-sheet. LCAT presents a Ser/Asp/His catalytic triad with a peculiar geometry, which is shared with such other enzyme classes as lipases, proteases and esterases. Our proposed model was validated through different approaches. We evaluated the impact on LCAT structure of some point mutations close to the enzyme active site (Lys218Asn, Thr274Ala, Thr274Ile) and explained, at a molecular level, their phenotypic effects. Furthermore, we devised some LCAT modulators either designed through a de novo strategy or identified through a virtual high-throughput screening pipeline. The tested compounds were proven to be potent inhibitors of the enzyme activity.

## Introduction

Protein members of the α/β hydrolase superfamily, present in all living organisms, share the same structural architecture but do not have common functions. This implies that the same fold has been used through evolution for a number of different functions including the catalytic activity as, for instance, hydrolase and esterase [1]. The canonical fold of this superfamily consists of an 8-stranded, mainly parallel, β-sheet surrounded by α-helices, in which the second strand is oriented in the antiparallel direction. No sequence similarity can be detected among the members of this superfamily [2]. LCAT (phosphatidylcholine-sterol acyltransferase, EC 2.3.1.43) belongs to the α/β hydrolase folding superfamily and shares the Ser/Asp-Glu/His triad with lipases, esterases and proteases, as already thoroughly discussed by Peelman et al. in 1998 [3].

The LCAT reaction consists in a trans-esterification, in which a fatty acid at the sn-2 position of phosphatidylcholine, or lecithin, is transferred to the free hydroxyl group of cholesterol, and in the meantime phosphatidylcholine is converted into lysophosphatidylcholine. However, at an atomic level, the mechanism is not yet accurately described [3].

LCAT catalyses the synthesis of most plasma cholesteryl esters (CE) [4], [5]. The preferred lipoprotein substrate for LCAT is a newly assembled small discoidal HDL and LCAT activity modulates its assembly [6].

Mutations in the *LCAT* gene cause two rare disorders, namely familial LCAT deficiency [7], FLD (MIM n. 245900) and fish-eye disease [8], FED (MIM n. 136120). In FLD, plasma LCAT is either absent or completely lacks catalytic activity; in FED, the mutant LCAT lacks activity on HDL lipids but esterifies cholesterol bound to apolipoprotein (apo)B-containing lipoproteins. In order to discriminate between FLD and FED in carriers of two mutant LCAT alleles, it is mandatory to measure the ability of plasma to esterify cholesterol; a differential diagnosis cannot be defined only from the molecular characteristics of the carriers.

Knowledge of LCAT atomic structure and identification of the amino acids relevant in controlling LCAT structure and function is expected to be very helpful in understanding its catalytic mechanism and its role in cholesterol metabolism. To date, the structure of LCAT has not been experimentally solved [9], [10]. The limiting step is represented by the enzyme purification from human plasma: LCAT is not present at a high concentration, and is strongly associated to lipoproteins. An alternative approach to obtain the LCAT atomic structure may be based on molecular modeling through up-to-date *in silico* procedures.

Modeling LCAT structure, however, faces a number of problems. LCAT lacks an appropriate template for a straightforward homology modeling: the protein has a very low sequence identity with all available templates, even if the secondary structure motives of α/β hydrolases are easy to recognize. In the past, Peelman et al. [3] tried to model the protein structure following a smart strategy: LCAT N−terminus (residues 73−210) was modeled on human pancreatic lipase and the active site (aa 333−399) was completed based on *C. antarctica* lipase structure; the remaining part of LCAT was not modeled.

In this paper, we can go further and propose a new LCAT atomistic model. It was built combining the most up-to-date *in silico* approaches and exploiting some crystallographic structures solved in recent years. Among the latter, we selected two protein structures useful to build the 3D LCAT model: PhaZ7depolymerase from *Paucimonas lemoignei* [11], and Lipase A from *Candida antarctica* [12]. The model was successfully validated in two ways. Some natural mutations were selected and structural details of the active site, of the catalytic triad and the oxyanion hole were used for a molecular explanation of their phenotypic effects. Then, we identified molecules able to inhibit LCAT enzymatic activity. These were either designed by a *de novo* strategy or identified through a virtual high-throughput screening pipeline; we could confirm their binding to the LCAT pocket by *in vitro* and *in vivo* activity assays.

## Materials and Methods

### Comparative Modelling

The human LCAT primary structure was downloaded from the UniProt-Protein Knowledgebase database (entry UniProt ID: P04180), and the signal peptide primary structure was removed. Starting from its sequence, a three-dimensional model was built based on multiple templates and ab initio modeling. Since a homologous template search on the full LCAT sequence did not provide any suitable solution, the LCAT primary structure was split in two parts, an N-terminal and a C-terminal ‘domain’. These domains do not correspond to true structural domains, but they were useful to find suitable templates for successful distant homology modeling procedures. We split the sequence in two portions of similar size, with a small overlapping region, suitable to drive domain merging. We also separated the catalytic triad, taking Ser181 in the N-terminal and Asp345 and His377 in the C-terminal parts: the triad interaction network would then assess the reliability of the model assembly procedure.

We separately submitted both LCAT parts to the Fold Recognition PSIPRED default procedure. We found 2VTV (PhaZ7depolymerase from *Paucimonas lemoignei*, UniProt ID: Q939Q9; identity: 19.5%) as a suitable template for LCAT N-terminus, and 2VEO (Lipase A from *Candida antarctica*, UniProt ID: D4PHA8; identity: 14.5%) as a template for LCAT C-terminus. All the modeling procedures were carried out with modules of the suite Molecular Operating Environment 2008.10 (MOE).

The alignment of the sequences of target and template proteins was produced with the Align program of MOE using default parameters, and it was manually adjusted making reference to the BLASTP output. This alignment was set as reference for all the homology modeling procedures.

Comparative model building was carried out with the MOE Homology Model program. 2VTV was set as template for LCAT residues 44–210 and 2VEO for residues 200–416. Ten independent models were built, refined and scored with GBIV scoring function, and then the highest-scoring intermediate model was submitted to a further round of energy minimization (EM). Both for the intermediate and the final structures the refinement procedures consisted in EM runs based on the AMBER99 forcefield, with the reaction field model, down to a gradient of 10^−5^ kcal/mol/Å^2^.

The two disulfide bonds of the protein, between Cys 50 and Cys 74, and between Cys 313 and Cys 356, were built through the MOE Builder module. Only nine structures had the cysteines of each pair sufficiently close to build of a disulfide bond; the best model had a distance of 5.5 Å for the pair Cys50–Cys74 and a distance of 5.18 Å for the pair Cys313–Cys356.

The quality of the final model was carefully checked with the MOE Protein Geometry module to make sure that the stereochemical quality of the proposed structure was acceptable. No further model quality estimations were run, because it is well-know that membrane or lipid-associated proteins, such as LCAT, obtain low scores, since their physico-chemical properties differ considerably from those of soluble proteins [13].

At the same time, we submitted the primary structure of LCAT to David Baker group’s Robetta Web Server, which uses the Rosetta software package, setting default parameters [14]. From the output, we selected the model with the correct general topology [3], the correct geometry of the catalytic triad [3], [15], and the most favourable rotamer conformations of side chains in order to form cysteine disulfide bridges.

From the above, once we obtained the two structures, that by comparative modeling and that by *ab initio* modeling, we built a ‘structural chimera’, setting the previous distant homology model as the template for the whole protein and the Robetta *de novo* model as template for residues 1–43; the option ‘Use Selected Residues to Override Template(s)’ was checked in order to override the primary template with the more appropriate ones only for the selected residues. Summing up, the residues from 1 to 43 were modeled *ab initio* by Robetta Server, the residues from 44 to 210 were modeled on 2VTV, and the residues from 211 to 416 were modeled on 2VEO. The modeling of residues from 1 to 91 has a low accuracy due to the combination of *ab initio* modeling and a low quality of the local alignment. All models were minimized and geometrically and energetically evaluated as already described above. Both disulfide bonds were set as described above. The LCAT binding site was identified through the MOE Site Finder module.

### Mutation Analysis

For all the selected residues, we performed the mutations through the MOE Mutate program in MOE Edit module, and we evaluated the best energetic orientation of side chains through the MOE Rotamer Explorer program in MOE Protein module.

For the Thr274 [Ala/Ile] mutations, we assessed the influence on protein unfolding free energy by using Protein Design module, a tool of MOE 2012.10 (Molecular Operating Environment, MOE). We refined the Thr274Ala and Thr274Ile mutations using the Protonate 3D MOE tool into the Protein Design applications, with default parameters and based on Amber12EHT forcefield with distance dependent dielectric form to model the solvent effects on electrostatics. Then we computed the ΔΔGs according to the following stability scoring function functional form:
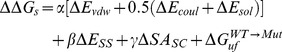
(1)where ΔE_vdw_ is the AMBER van der Waals interaction energy, ΔE_coul_ is the AMBER Coulomb interaction energy, ΔE_sol_ is the change in solvation energy calculated using GBVI, and E_SS_ is the change in energy due to the presence of a disulfide bond. The last term, γ·ΔSA_sc_ is a residue-dependent change in surface area (associated to entropy). We also generated an ensemble of protein conformations for both the Thr274 mutants using MOE LowMode MD with default parameters, and based on Amber12EHT forcefield, with distance dependent dielectric form to efficiently search for the conformational space of the wild type and of the mutant protein forms.

In detail, for the generated ensemble, we evaluated Stability, the absolute thermostability of the mutation, which is equal to the Boltzmann average of the stabilities of the ensemble, and dStability, the relative thermostability of the mutation with respect to the wild-type protein, computed as Boltzmann average of the relative stabilities of the ensemble [16], [17]. A ΔΔG_s_ negative value points to a mutation able to stabilize the protein; on the contrary a positive ΔΔG_s_ suggests a mutation, which reduces the global protein stability.

### Molecular Docking

The Asinex Platinum Collection (http://www.asinex.com) is a lead-like structural library containing approx. 130,000 in-house synthesized compounds. The SD file containing all the structures was downloaded and the MOE Conformation Import module was run on this file to produce a single, low-energy conformation for each putative ligand contained in the Asinex SD file. All the docking procedures were carried out with the suitable MOE programs.

The *in silico* screening was carried out with the Dock program contained in the MOE Simulation module. The full LCAT structure was set as Receptor. The binding site was defined with dummy atoms positioned through the MOE Site Finder module. Before starting with the placement procedure, 1000 conformations were generated for each ligand by sampling their rotatable bonds. The selected placement methodology was Triangle Matcher, in which the pre-refined poses are generated by superposing triplets of ligand atoms and triplets of receptor site points. The protein site points are alpha spheres centres that represent locations of tight packing. Before scoring all the generated poses, duplicate complexes were removed. Poses are considered as duplicates if the same set of substrate-enzyme atom pairs are involved in hydrogen-bond interactions and the same set of ligand atom-protein residue pairs are involved in hydrophobic interactions. The accepted poses were scored according to the London dG scoring, which estimates the free energy of binding of the ligand from a given pose:
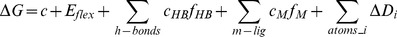
(2)where *c* represents the average gain/loss of rotational and translational entropy; *E_flex_* is the energy due to the loss of flexibility of the ligand (calculated from ligand topology only); *f_HB_* measures geometric imperfections of hydrogen-bonds and takes a value in [0,1]; *c_HB_* is the energy of an ideal hydrogen-bond; *f_M_* measures geometric imperfections of metal ligations and takes a value in [0,1]; *c_M_* is the energy of an ideal metal ligation; and *D_i_* is the desolvation energy of atom *i*. The difference in desolvation energies is calculated according to:
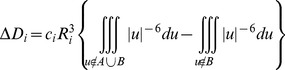
(3)where *A* and *B* are the protein and/or ligand volumes with atom *i* belonging to volume *B*; *R_i_* is the solvation radius of atom *i* (taken as the OPLS-AA van der Waals sigma parameter plus 0.5 Å ); and *c_i_* is the desolvation coefficient of atom *i*. The coefficients (*c, c_HM_, c_M_, ci*) have been fitted from approx. 400 X-ray crystal structures of protein-ligand complexes with available experimental pKi data. Atoms are categorized into about a dozen atom types for the assignment of the *c_i_* coefficients. The triple integrals are approximated using Generalized Born integral formulas. Only the top scoring solution was kept and submitted to a further refinement step, via molecular mechanics (MM) based on MMFF94x. In order to speed up the calculation, residues over a 6 Å cut-off distance away from the pre-refined pose were ignored, both during the refinement and in the final energy evaluation. All receptor atoms were held fixed during the refinement. During the course of the refinement, solvation effects were calculated using the reaction field functional form for the electrostatic energy term. The final energy was evaluated using the MMFF94x forcefield with the Generalized Born solvation model (GBIV) [18]. All the ligands contained in the Platinum library were screened according to the above procedure. Once sorted by their docking score, we selected the best docking poses and only the two top scoring compounds were resubmitted to the same docking procedure, keeping for each of them 300 poses. Both were eventually bought from Asinex and tested in *in vitro* assays.

We designed an irreversible inhibitor, connecting a molecule of cholesterol through a phosphonyl chloride group to a 17-carbon atom chain. We evaluated *in silico* both its enantiomers, R and S, for interaction with LCAT by performing a molecular docking, using the Dock program in the MOE Simulation module. After generating 1000 conformations, we produced 100 poses for both molecules using the AlphaPMI placement methodology, useful for docking to a tight pocket. The accepted poses were assessed according to the London dG score and refined through a MM step based on MMFF94x with the GBIV solvation model. We selected the best poses for R and S enantiomers and, using the Binding tool in MOE suite, we covalently bound *in silico* both molecules to the catalytic serine of LCAT. We then performed an EM step, based on MMFF94x with the GBIV solvation model, down to a gradient of 10^−5^ kcal/mol/Å^2^. The accurate docking procedure described above for the chemical library items was applied to the *de novo* designed compound.

The estimated binding affinities were calculated through the MOE LigX module. The prediction of pKi values (-Log of the dissociation constant) was computed through the London dG scoring function and the Lig X MOE module, after a further local refinement of the docked complexes into the LCAT active site. This and other empirical scoring functions are useful to rank the complexes according to their dissociation constant, as already discussed by Eberini et al. [19].

### *In vitro* Assays

Plasma obtained from a control subject was added with increasing concentrations (0.046–6.620 mM) of LCAT modulators or saline and cholesterol esterification rate (CER) was assessed by measuring changes in plasma unesterified cholesterol concentrations [20].

The ability of the LCAT irreversible modulator to bind recombinant human LCAT [21] was tested by mass spectrometry. Molecular weight was determined by a Bruker Daltonics Reflex IV instrument (Bruker Daltonics, Bremen, Germany) equipped with a nitrogen laser (337 nm) and operated in positive mode using sinapinic acid in 01% TFA: CH3CN = 2∶1 as matrix. External standards were used for calibration (Bruker protein calibration standard).

### *In vivo* Assays

Animal care and experimental procedure were performed with the approval of the local “Comitato Etico per la Sperimentazione Animale”, overseeing animal experiments at University of Parma. No special permission for use of animals (mice) in such pharmacological studies is required in Italy, as defined by the legislative decree 116/92.

Twelve week old male C57BL/6J mice were housed in a controlled environment at 25±2°C with alternating 12 h light and dark cycles and received standard diet and water ad libitum. Mice (n = 5) were treated intraperitoneally with the covalent inhibitor at a dose of 150 mg/kg. Blood samples were collected at different times after treatmentand recovered in plastic tubes containing sodium citrate 3.8%. Plasma was isolated by low speed centrifugation and stored at −80°C until use. Plasma total and unesterified cholesterol were measured by enzymatic techniques, and LCAT activity was measured on plasma samples using reconstituted HDL as substrate [22].

## Results and Discussion

### Comparative Modelling

The PDB Search module of the MOE Suite was unable to identify any suitable template(s) for full LCAT modeling. Also submitting the entire LCAT sequence to the Fold Recognition of PSIPRED server [23] did not identify any useful entry, and the best matching protein with a solved structure was a hydrolase from *Lactobacillus plantarum* (PDB ID 3LP5, UniProt ID: F9UMW5). However, the latter shares with LCAT only 16% identity - a level of similarity insufficient to produce any acceptably accurate model. All the identified templates belong to the fold superfamily of α/β-hydrolases, confirming previous hypotheses that LCAT belongs to it. Scanty results were likewise obtained by using the template identification tool of the Swiss-Model web site (http://swissmodel.expasy.org), which carries out a multi-level search based also on Hidden Markov Models (HMM). This tool is not based only on the search for identity, but is sensitive enough to detect distant relationships among protein families [24]. The application of homology modeling procedures can produce very reliable and useful results also when the identity between target and template sequences is very low (approx. 20%). If the general topology and the secondary structure of the target protein is known, the alignment procedure can be carried out more confidently through the use of these supplementary data. Typical example is the modeling of class-A GPCR [25], receptors with a peculiar topology, that can be easily managed through classical comparative methods, despite their low sequence identity with the available templates. Recently, we focused our attention on modeling of GPR17, a class-A GPCR, and successfully identified very potent orthosteric agonists of this receptor [19]. LCAT topology and local secondary structure have been thoroughly discussed and are well-defined [3]. These data are very useful for guiding a better alignment between target and template sequences, and for obtaining a more reliable three-dimensional model.

An effective strategy to try overcoming the lack of a single whole suitable template is building homology models of distinct parts of a protein, and then merging them in a single model that can be defined as a ‘structural chimera’ [19]. Indeed, we had better success after splitting the LCAT primary structure approx. in two halves, which allowed the identification of two templates useful to carry out LCAT distant homology modeling, as already suggest by Peelman et al. [3]. As reported in the ‘Materials and Methods’ section, to guide merging we identified a partially overlapping portion (residues 200–210), thus splitting the catalytic triad between the two ‘domains’ (Ser181 in the N-terminal, and Asp345 and His377 in the C-terminal domain). Submitting the N-terminal (residues 1–210) and C-terminal (residues 200–416) LCAT sequences, we obtained two series of putative templates, with higher identity to the targets than *Lactobacillus plantarum* hydrolase, but still under 30%.

In detail, as template for LCAT N-terminus, we found 2VTV (PhaZ7 depolymerase from *Paucimonas lemoignei*, UniProt ID: Q939Q9. Identity: 19.5%) and, for LCAT C-terminus, 2VEO (lipase A from *Candida antarctica*, UniProt ID: D4PHA8. Identity: 14.5%) (see Figure S1 in File S1).

The final comparative model matched the already reported topology [2], [3], in which we can identify the combination of α-helices connected by variable loops and covering a central β-sheet. In detail, the model presents the correct arrangement for the seven strands in the central β-sheet (β2-β4-β3-β5-β6-β7-β8), and their correct relative orientation, since β2 has antiparallel orientation with respect to the other strands. The localization of the catalytic residue Ser181 at the end of the β-strand 5, close to α-helices 5 and 6, is in line with previously published data.

In order to collect more knowledge about LCAT structure and to manage these structural issues, we thus produced, in addition, a *de novo* model of LCAT, by submitting to this purpose the primary structure of LCAT, without its signal peptide, to the Robetta Web Server. The *de novo* approaches to protein modeling have been extensively analysed and evaluated in the most recent CASP competitions [26], demonstrating that there are several cases in which Rosetta has been able to predict structures with atomic level accuracy better than 2.5 Å [27].

Only five of all *de novo* generated models had the correct general topology; two of these lacked the correct geometry of the catalytic triad, and were discarded without further investigations. In the three remaining models, the possibility to form cysteine disulfide bridges was evaluated: the C-terminal cystine was correctly predicted in all of them, whereas no acceptable predictions were obtained for the N-terminal cystine. Looking into the catalytic triad, one of the three models presented a very favourable interaction network and relative distances among the three residues. Furthermore, also the localization of the oxyanion hole was correctly predicted: this residue contributes to the stabilization of the reaction intermediate [28] and is spatially close to the catalytic triad residues. We selected this one as the best model obtained from the *de novo* Robetta strategy.

In order to keep into account all structural information about LCAT, we merged the homology and the *de novo* models in a new final LCAT model, by adding to the homology model (residues 44–416) the residues 1–43 obtained from the *ab initio* modeling procedure. The final model of LCAT is reported in Figure S2 in File S1.

The catalytic triad of our proposed model appears correctly predicted, when compared with the model proposed by Peelman et al. [3], and with the model catalytic triad of a serine protease from *Bacillus lentus* [15]. We also found general agreement of the distances between the catalytic residues in our model with those reported by Peelman et al. [3]: the distance between Oγ in Ser181 and N2 in His377 was 5.41 Å *versus* an expected distance of 2.5 Å, the distance between Oδ in Asp345 and N1 in His377 was 4.63 Å *versus* an expected distance of 2.9 Å, the distance between the oxyanion hole (Phe103) and the catalytic triad was 6.75 Å *versus* an expected distance of 5 Å.

LCAT, which has both phospholipasic and acyltransferasic activities, requires the possibility for both a lecithin and a cholesterol molecule to enter the catalytic site. For this reason, we expected to find in the LCAT structure a hydrophobic pocket large enough to accommodate a CE, with an accessible catalytic triad placed at its basis (active site), and not completely solvent-exposed.

We used the MOE Site Finder tool to identify the binding site for LCAT natural ligands. The analysis of LCAT through the MOE Site Finder module revealed 27 putative binding sites; the top-scoring contained 346 contact atoms, among which 73 were hydrophobic and 256 corresponded to side-chain atoms. Since this pocket, shown in Figure S2 in File S1, is located near the catalytic triad, we accept this one as the putative LCAT binding site.

The scientific literature reports that mutation of Glu149 changes the fatty acid specificity and probably facilitates the spatial accommodation of a bulkier arachidonic acid molecule in the binding site [3], [29]. As mapped on our model, Glu149 is localized, as expected, in the loop between helices 4 and 5. It does not directly faces the active site, but has a pivot role in a network of hydrogen bonds with amino acids relevant for the binding site structure. The loss of Glu149 side chain breaks this structural network and makes the active site looser, as reported in Figure S3 in File S1.

In most lipases, it has been reported that a mobile lid covers the substrate binding site, and enzyme activation occurs upon binding to a hydrophobic substrate. In detail, in water, a lid closes the entering channel for the active site, which opens only upon binding to a hydrophobic interface. Usually, the lid consists of a single short helix, and it is closed onto the active site. In contrast to the core of lipases, whose architecture is highly conserved, lids are less conserved elements, with significant variations in their length and different relative positions in the various lipases. In LCAT, several experiments have pointed out an amphipathic region: an α-helix in the N-terminus, closed, as in other lipases, by a disulphide bridge (Cys50–Cys74) [10]. This region is identified as an interfacial recognition domain for (apo)lipoproteins, and it could not only serve as a binding site for the hydrophobic substrates, but further include a ‘tilted’ peptide, which is thought to destabilize the lipid substrate and facilitate the diffusion of a monomeric phospholipid or triglyceride into the cavity of the enzyme active site [30]. This region is distant from the enzyme active site, which is located in the central core of the protein. Contrary to the latter, which is more conserved and easier to model, in our LCAT structure we then cannot properly define the N-terminal lid region, because it is not sufficiently conserved. Furthermore, the lid region requires a hydrophobic environment for a complete and correct folding, and MM computations accounting for a high and continuous dielectric effect are inadequate to correctly model this part of the protein [29], [30]. As already discussed, the LCAT N-terminus is associated with very low accuracy, connected to the lack of homologous templates for the residues from 1 to 43 and to a low quality of the local alignment with the selected template for the region from 44 to 91.

### Mutation Analysis

A validation of the structure suitability comes from the mapping of known mutations, which cause familial LCAT deficiency [7], FLD (MIM n. 245900), and fish-eye disease [8], FED (MIM n. 136120), both characterized by very low levels of HDL [31]. We selected three mutations described in Italian carriers of LCAT deficiency [31], Lys218Asn, Thr274Ala and Thr274Ile, and evaluated their impact on the atomic structure [32], [33].

When analysing the LCAT primary structure the impact of the listed mutations is not obvious, because these amino acids are not located in the immediate neighbourhood of the catalytic residues nor directly affect them.

Mutation of Lys218 in Asn results in FLD in homozygous carriers [31]. Modeling the entire enzyme and locating this mutation on the LCAT three-dimensional structure, we find out that the residue faces the active site, in the region that we assume to behave as fatty acid cleft. This mutation modifies the environment, since the residue changes from a charged (basic) to a non-charged amino acid. In addition, we observe that this mutation produces the disruption of the local hydrogen-bond network. These changes strongly affect the fatty acid binding site and may explain why the carriers of this mutation do not have a functional enzyme and cannot produce cholesterol esters (see Figure S4 in File S1).

Two different mutations for Thr274 have been reported [31]; Thr274Ala results in FED and Thr274Ile in FLD. Also in this case, the atomic structure of LCAT helps us to explain the different impact of the two mutations and to assess the quality of the model.

As reported in Table 1, the three structures, wild-type and mutated, have different stability scores. The Ala mutation results in a negligible decrease in stability (dStability = 0.2696 kcal/mol), whereas the Ile mutation considerably increases global protein stability (dStability = −2.8646 kcal/mol).

**Table 1:** Stability score of WT and T274 mutant LCAT.

Mutation	Stability* [kcal/mol]	dStability** [kcal/mol]
T274 (WT)	−7.37	0.00
T274A	−6.12	0.27
T274I	−9.25	−2.87

*Stability is the absolute thermostability of the mutation and, for the generated ensemble, it is equal to the Boltzmann average of the stabilities of the ensemble.

**dStability is the relative thermostability of the mutation in comparison with the wild-type protein, and it is equal to the Boltzmann average of the relative stabilities of the ensemble.

Thr274 is close to Phe103, which contributes to the definition of the oxyanion hole (2.5 Å), and its mutation into Ala increases the environment hydrophobicity. We can assume that the local conformational rearrangement due to the mutation results in a change in the Phe103 side chain orientation, causing a difficulty in enzyme-substrate binding and the occurrence of the FED phenotype. The Ile residue is associated with a bigger steric bulk than Ala, and this can cause a wider local rearrangement, moving the Phe103 side chain almost completely into the binding pocket, thus blocking LCAT enzymatic activity.

**Figure 1:**
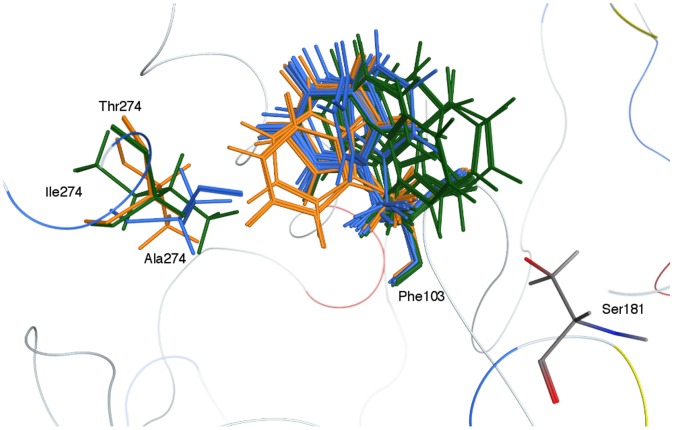
Superposition of the top 10 conformations obtained by LowMode MD for wild-type LCAT and T274[A/I] mutants. Protein backbone is rendered in ribbons, whereas Phe103, Ser181 and Thr274[Ala/Ile] side chains are rendered as sticks. Color code: wild-type LCAT = orange, T274A = blue, T274I = green.

To validate these hypotheses, we performed a conformational search on the wild-type and on the mutated structures; using the LowMode MD method, we produced 25 different poses for each structure and we analysed the 10 more energetically favourable conformations. As reported in Figure 1, the T274A mutation (in blue) causes a minimal change in Phe103 orientation *versus* the wild-type molecule. Conversely, the T274I mutation (in green) produces a more considerable rearrangement, and 4 structures out of the 10 analysed present the Phe103 side chain in a position very close to the catalytic serine, and a strong decrease of the binding area. Indeed, no room remains for the stabilization of the tetrahedral intermediate typical of a trans-esterification reaction, and carriers of this mutation present FLD phenotype.

### Molecular Docking

Searching for molecules targeting the LCAT binding site in a large chemical library, we carried out the docking procedure in two steps: i) quick docking and ii) accurate docking. To evaluate the docking results in the final step of both procedures, we run molecular mechanics refinement, and computed the final energy score through an empirical scoring function based on the MMFF94x forcefield with the generalized Born implicit solvation model (MM/GBIV).

All ligands in the tested Asinex database were submitted to a virtual HTS (high-throughput screening) procedure and were evaluated using the quick docking procedure, refining and keeping only the best solution for each ligand. The ligands corresponding to the two best poses were submitted to the accurate docking procedure, generating 300 solutions (poses) for each ligand. The best solution for the ligands shows binding scores between −7.356 and −7.289 kcal/mol, as reported in Table 2.

**Table 2:** Physico-chemical parameters for the two top-scoring ligands.

Ligand	MM/GBIVdocking score [kcal/mol]	Affinity (pKi)
Compound #1	−7.36	5.84
Compound #2	−7.29	5.39

**Figure 2:**
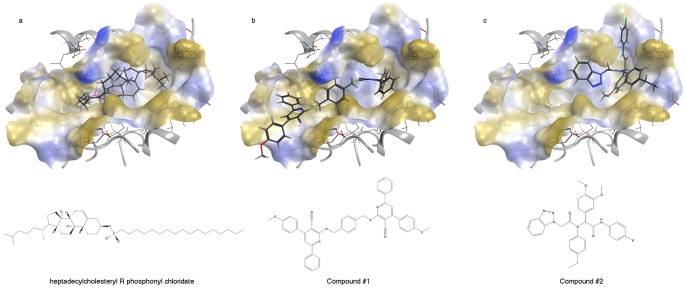
Molecular docking results: a) lowest energy pose in LCAT binding site for heptadecylcholesteryl R phosphonyl chloridate b) compound #1 and c) compound #2 and its chemical structures. The surface of the protein binding site is colored according to lipophilicity (hydrophilic area in blue in grey, lipophilic in gold and neutral in white).

The chemical structures of the two top-scoring compounds are reported in Figure 2, panel b and c. They belong to different chemical classes, suggesting that the *in silico* screening allowed us to identify putative lead compounds with different features. The *in silico* simulation provides evidence that both molecules completely block the access to the LCAT active site, as shown in Figure 2, panel b and c.

The docking score of the poses according to MM/GBIV and the pK_i_ values (-Log(K_i_)), dissociation constant) computed with the London dG scoring function after MOE LigX refinement show a similar trend, suggesting that both these methods, based on different scoring approaches, can be used to evaluate docking results and to compute approximate binding free energies for the system under investigation (see Table 2).

With the structural model of LCAT as reference, we were able to design in addition a compound capable of acting as irreversible inhibitor of the enzyme. Such a molecule may be potentially useful as a tool in pharmacological research.

To design an inhibitor with a highly selective profile, we set to mimic the II intermediate of the reaction catalysed by the enzyme - a compound in which a cholesterol and a fatty acid molecule are bound together. Indeed, the I reaction intermediate could be common to other enzymes that share the same catalytic triad, and this would certainly result in a loss of selectivity [34].

For this reason, we designed a compound joining a molecule of cholesterol bearing a phosphonyl chloride group to a 17-carbon atom chain, an optimal length for the functionality of LCAT [35]. This resulted in the synthesis of a heptadecylcholesteryl-(R, S)-phosphonyl chloridate, a compound that is able to fully occupy the active site of LCAT, as shown by our simulation, see Figure 2, panel a [36]. Since this molecule has a chiral centre (the phosphorus atom), both enantiomers were tested *in silico*. After docking both the R and S compounds, and after covalently linking Ser181Oγ to the phosphorus atom in the top-scoring complexes of the two lists, we submitted them to a further energy minimization step. The R-enantiomer complex turned out to have a more favourable potential energy (−2489.68 kcal/mol), whereas the S-enantiomer has a potential energy of −2130.58 kcal/mol. Furthermore, observing the binding site, only the R-enantiomer seems to be properly placed and oriented for interaction with the oxyanion hole, close to the Phe103 residue. A similar enantiomeric preference had already been described for some lipases that share the same catalytic triad as LCAT [37].

Even though the computational data suggest that the R-enantiomer is stereochemically and energetically favoured (please, see before), we used the readily available racemic mixture for the *in vitro* tests of LCAT enzymatic activity. In order to compare the complementarity of the irreversible inhibitor *versus* the reversible ones with respect to LCAT, the non-covalent interaction energies (disregarding the covalent bond) were computed *in silico* by using the reported molecular docking protocol; the binding score had a value of −8.49 kcal/mol.

The ability to affect the cholesterol esterification process of the identified LCAT inhibitors (both the HTS molecules and the covalent inhibitor) was assessed *in vitro* by the measurement of CER in control human plasma in the absence and presence of increasing amounts of the modulators. Each series of tests was repeated three times; the results are reported in Table 3. All the molecules are able to inhibit the enzyme in a dose-dependent manner, although at different concentrations. The most efficacious compound among the selected molecules completely inhibits LCAT at a concentration between 0.364 to 0.730 mM, while the heptadecylcholesteryl-(R, S)-phosphonyl chloridate does it at 6.620 mM. Data have been plotted in Figure S5 in File S1.

**Table 3:** *In vitro* inhibitory assays on LCAT of the heptadecylcholesteryl (R, S) phosphonyl chloridate and the two top-scoring compounds.

Inhibitor concentration [mM]	heptadecylcholesteryl (R, S) phosphonyl chloridate % inhibition	Compound #1 % inhibition	Compound #2 % inhibition
0.046	0.00	25.3±4.1	30.2±0.0
0.091	0.00	69.3±5.1	36.1±4.2
0.182	0.00	41.6±2.3	45.3±8.0
0.364	0.00	88.8±6.8	51.6±2.7
0.730	16.6±8.8	100±0.0	100±0.0
1.420	33.8±4.6		
2.680	54.6±5.2		
6.620	100±0.0		
**Approx. IC50**	2.1	0.1	0.3

The only compound designed to irreversibly inhibit LCAT is heptadecylcholesteryl (R, S) phosphonyl chloridate, because it has acylating activity. In order to demonstrate that this compound is able to acylate the catalytic Ser residue, we have carried out MALDI-TOF analysis of the recombinant human LCAT [21] before and after incubation with heptadecylcholesteryl (R, S) phosphonyl chloridate. Through this approach, we demonstrated the formation of a covalent bond between LCAT and the inhibitor. Peaks at 57800.9 m/z and at 58445.6 m/z were detected before and after LCAT incubation: the first one corresponds to the free glycosylated enzyme, the latter to LCAT bound to the inhibitor (Table 4).

**Table 4:** Mass spectrometry data of human recombinant LCAT and of the covalent adduct between LCAT and its irreversible inhibitor.

	MW (calculated)* (Da)	MW (experimental) (Da)
LCAT	47083.9	57800.9
LCAT+inhibitor	47726.9 (+643)	58445.6 (+644.7)

*Molecular weight calculated from the aminoacid sequence. An increase of 643 Da is expected in the presence of one molecule of inhibitor bound to the protein. The higher MW experimentally observed for LCAT in comparison with the value calculated from the sequence can be ascribed to the glycosylation of the protein.

When the heptadecylcholesteryl-(R, S)-phosphonyl chloridate was injected intraperitoneally in mice, it inhibited LCAT activity in plasma by 36% after 30 hours and by 100% 45 hours after injection (LCAT activity at baseline: 3.6 nmol CE/ml/h ±2.8), and free/total cholesterol ratio increased from 0.35±0.02 to 0.41±0.06 and 0.39±0.06 at 30 h and 45 h, respectively.

## Conclusions

Our *de novo* design of an irreversible inhibitor is based on the hypothesized LCAT catalytic mechanism. Analysing the docking results, we could recognize two distinct pocket portions able to bind: i) the cyclopentanoperhydrophenanthrene nucleus and ii) the long-chain fatty acid, as schematically reported in Figure 2, panel a. As previously mentioned, both these regions are strongly hydrophobic. The demonstration that some of the identified molecules are able to bind LCAT suggests that our distant comparative modeling strategy allowed us to predict the shape of the enzyme active site at a satisfactory approximation level.

A well-developed body of knowledge assigns to LCAT a central role in intravascular HDL metabolism and in the determination of plasma HDL levels. Knowledge of LCAT atomic structure is expected to be very helpful in understanding its catalytic mechanism and investigating its effect on atherogenesis. We believe that our results show that the three-dimensional LCAT model we generated represents a useful tool for the study of its poorly characterized catalytic mechanism and, in perspective, for the design of chemicals targeting LCAT active site.

## Supporting Information

File S1 **This file is organized in: Figure S1.** Alignment between: A) 2VTV2 and LCAT N-terminal part, B) 2VEO and LCAT C-terminal part, used during the modeling procedures, color-coded by similarity (BLOSUM62). **Figure S2.** LCAT 3D model and its binding site. Protein structure is rendered with ribbons and colored by modeling approach: residues from 1 to 43 in yellow (*ab initio* model), residues from 44 to 91 in orange (homology model on 2VTV, low quality), residues from 92 to 200 in red (homology model on 2VTV, high quality), residues from 200 to 211 in purple (homology model on 2VTV and 2VEO), and residues from 212 to 416 (homology model on 2VEO). The surface of the protein active site is colored according to CPK colors (carbon in grey, oxygen in red and nitrogen in blue). **Figure S3**. Interaction network of Glu149. Protein backbone is rendered in ribbons, whereas residues’ side chains are rendered as sticks. **Figure S4**. Interaction network of Lys 218. Protein backbone is rendered in ribbons, whereas residues’ side chains are rendered as sticks. **Figure S5**. Dose-response curves for the activity of a) compound #1, b) compound #2 and c) heptadecylcholesteryl-R-phosphonyl chloridate.
(DOCX)
