# Supplementary material for: Distant Homology Modeling of LCAT and Its Validation through In Silico Targeting and In Vitro and In Vivo Assays
Source: PLoS One. 2014 Apr 15;9(4):e95044. doi: 10.1371/journal.pone.0095044 (PMC3988154; doi:10.1371/journal.pone.0095044)
Supplement: File S1 — This file is organized in: Figure S1. Alignment between: A) 2VTV2 and LCAT N-terminal part, B) 2VEO and LCAT C-terminal part, used during the modeling procedures, color-coded by similarity (BLOSUM62). Figure S2. LCAT 3D model and its binding site. Protein structure is rendered with ribbons and colored by modeling approach: residues from 1 to 43 in yellow (ab initio model), residues from 44 to 91 in orange (homology model on 2VTV, low quality), residues from 92 to 200 in red (homology model on 2VTV, high quality), residues from 200 to 211 in purple (homology model on 2VTV and 2VEO), and residues from 212 to 416 (homology model on 2VEO). The surface of the protein active site is colored according to CPK colors (carbon in grey, oxygen in red and nitrogen in blue). Figure S3. Interaction network of Glu149. Protein backbone is rendered in ribbons, whereas residues’ side chains are rendered as sticks. Figure S4. Interaction network of Lys 218. Protein backbone is rendered in ribbons, whereas residues’ side chains are rendered as sticks. Figure S5. Dose-response curves for the activity of a) compound #1, b) compound #2 and c) heptadecylcholesteryl-R-phosphonyl chloridate. (DOCX) [file pone.0095044.s001.docx]

**Supporting Information**

**Distant homology modeling of LCAT and its validation through *in silico* targeting and *in vitro* and *in vivo* assays**

Cristina Sensi, Sara Simonelli, Ilaria Zanotti, Gabriella Tedeschi, Giulia Lusardi, Guido Franceschini, Laura Calabresi, and Ivano Eberini

^^

**Figure S1**. Alignment between: A) 2VTV2 and LCAT N-terminal part, B) 2VEO and LCAT C-terminal part, used during the modeling procedures, color-coded by similarity (BLOSUM62).


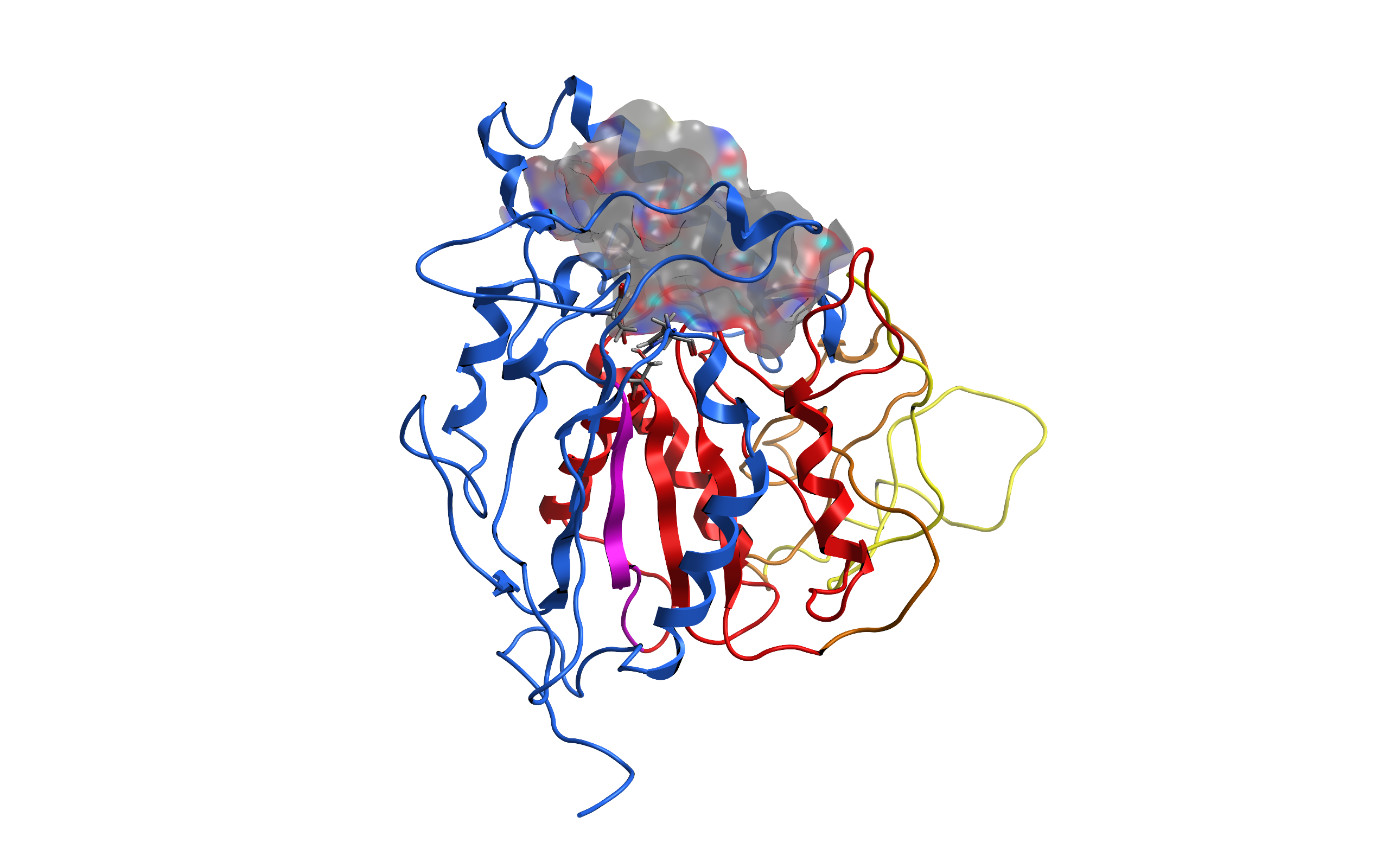


**Figure S2.** LCAT 3D model and its binding site. Protein structure is rendered with ribbons and colored by modeling approach: residues from 1 to 43 in yellow (*ab initio* model), residues from 44 to 91 in orange (homology model on 2VTV, low quality), residues from 92 to 200 in red (homology model on 2VTV, high quality), residues from 200 to 211 in purple (homology model on 2VTV and 2VEO), and residues from 212 to 416 (homology model on 2VEO). The surface of the protein active site is colored according to CPK colors (carbon in grey, oxygen in red and nitrogen in blue).

**
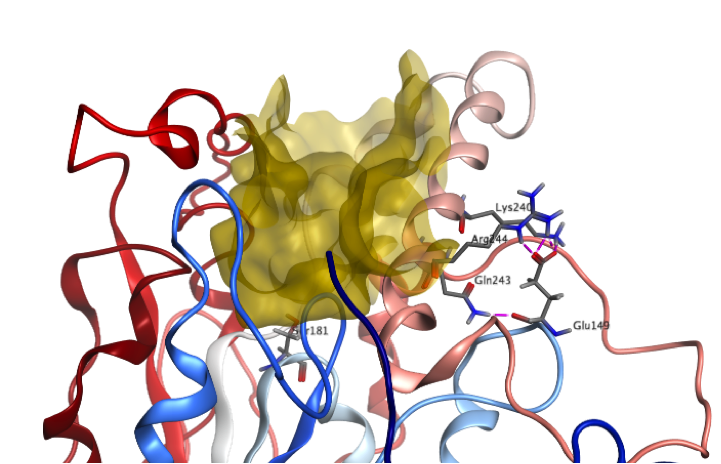
**

**Figure S3**. Interaction network of Glu149. Protein backbone is rendered in ribbons, whereas residues’ side chains are rendered as sticks.

**Figure S4**. Interaction network of Lys 218. Protein backbone is rendered in ribbons, whereas residues’ side chains are rendered as sticks.


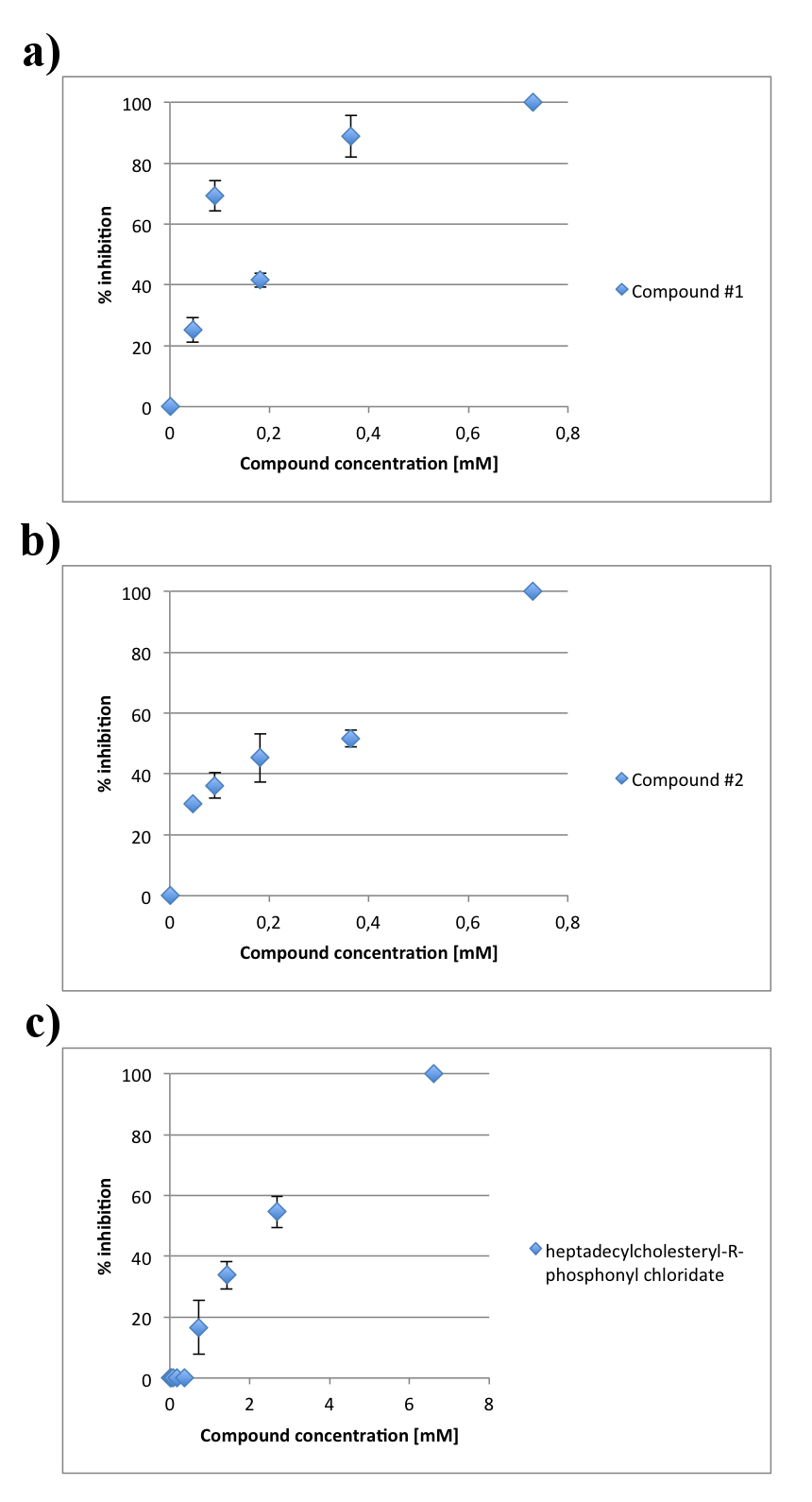


**Figure S5**. Dose-response curves for the activity of a) compound #1, b) compound #2 and c) heptadecylcholesteryl-R-phosphonyl chloridate
